# Ecological processes influencing bacterial community assembly across plant niche compartments

**DOI:** 10.1002/mlf2.70019

**Published:** 2025-06-24

**Authors:** Nazish Roy, Seongeun Yang, Dongmin Lee, Kihyuck Choi

**Affiliations:** ^1^ Department of Applied Bioscience Dong‐A University Busan Republic of Korea

**Keywords:** community assembly, ecological process partitioning, neutral theory, niche theory, sink–source analysis

## Abstract

Understanding microbial community assembly in plants is critical for advancing agricultural sustainability. This study investigated microbial diversity and community assembly mechanisms across six compartments of tomato plants: bulk soil, rhizosphere, root, stem, flower, and seed. Using 16S rRNA amplicon sequencing, we observed that microbial richness was highest in the bulk soil and rhizosphere, with significant reductions in internal plant tissues. Co‐occurrence network analysis identified distinct microbial hubs in each compartment, such as *Bacillus* in the root and seed, highlighting critical interactions influencing microbial dynamics. Ecological process modeling revealed that deterministic processes, such as selection, dominated in below‐ground compartments, whereas stochastic processes like drift were more influential in above‐ground tissues, reflecting differences in niche specificity and ecological stability. Dispersal limitation emerged as a key driver in soil‐associated compartments, structuring microbial diversity. These findings advance our understanding of the ecological mechanisms shaping plant microbiomes and suggest targeted microbiome management strategies to enhance crop health, productivity, and resilience. Future research integrating functional genomics, temporal dynamics, and environmental factors is necessary to uncover the broader implications of plant‐associated microbiomes.

## Impact statement

Plants host helpful microbes that can boost their growth and protect them from stress. This study explored how different parts of a tomato plant, like roots, flowers, and seeds, get their unique sets of bacteria. We found that some microbes are passed from the soil, while others come from the plant itself or even pollinators. Our findings show how nature and chance together shape these microbial communities. This knowledge can help farmers manage plant microbes in smarter ways, leading to healthier crops and more sustainable farming.

## INTRODUCTION

The plant microbiome, often considered an extension of the host genome, significantly influences plant health, stress tolerance, and productivity. This interaction is mediated by a complex community of microorganisms that directly affect the plant phenotype^1^, ^2^, ^3^. Tomato (*Solanum lycopersicum*), a crop of global economic importance, serves as a powerful model for microbiome research due to its well‐characterized genetics and diverse microbial associations^4^, ^5^, ^6^.

Community assembly in microbial ecosystems is governed by both deterministic (e.g., selection) and stochastic (e.g., dispersal, drift [DR]) processes, as described in Vellend's synthesis (2010) and other ecological frameworks^7^, ^8^. In plant systems, this complexity is amplified by interactions across multiple trophic levels. However, focusing on microbial communities as a single trophic level allows for clearer insights into these mechanisms^9^.

Different plant compartments (e.g., roots, rhizosphere [RH], stem, flower, and seed) act as distinct ecological niches, hosting unique microbial communities^10^, ^11^. Although numerous studies have investigated microbial communities in specific tomato plant compartments, such as the RH, phyllosphere, and endosphere, a holistic perspective that integrates the ecological assembly of microbiota across all major compartments remains absent^12^, ^13^, ^14^. In this study, we analyzed microbial community structure and assembly processes in six tomato plant compartments. We hypothesize that deterministic processes dominate in below‐ground compartments due to strong selection pressures, while stochastic processes are more prominent above ground. We also expect dispersal limitation (DL) to shape RH communities and DR to influence microbial diversity in aerial tissues. Using 16S rRNA sequencing and ecological modeling, we quantify the roles of selection, dispersal, and DR in shaping microbial communities. Our findings bridge a critical knowledge gap and establish a framework for microbiome‐based crop management strategies.

## RESULTS

To explore the microbial diversity within various compartments of the tomato plant, we analyzed the bacterial communities from the bulk soil, RH, root, stem, flower, and seed compartments (*n* = 10 per compartment). A total of 6,535,781 raw reads were generated by amplification of V3–V4 regions of the 16S rRNA gene. Reads were quality‐filtered (*Q* score > 25), denoised, and truncated at 290 and 220 bp. After denoising, operational taxonomic unit (OTU) clustering (97%), and removing singleton using divisive amplicon deconvolution algorithm (DADA2) and vector search engine for rapid identification and clustering of sequences (VSEARCH), 2765 OTUs were obtained.

### Microbial diversity varies across tomato plant compartments

**Figure 1:**
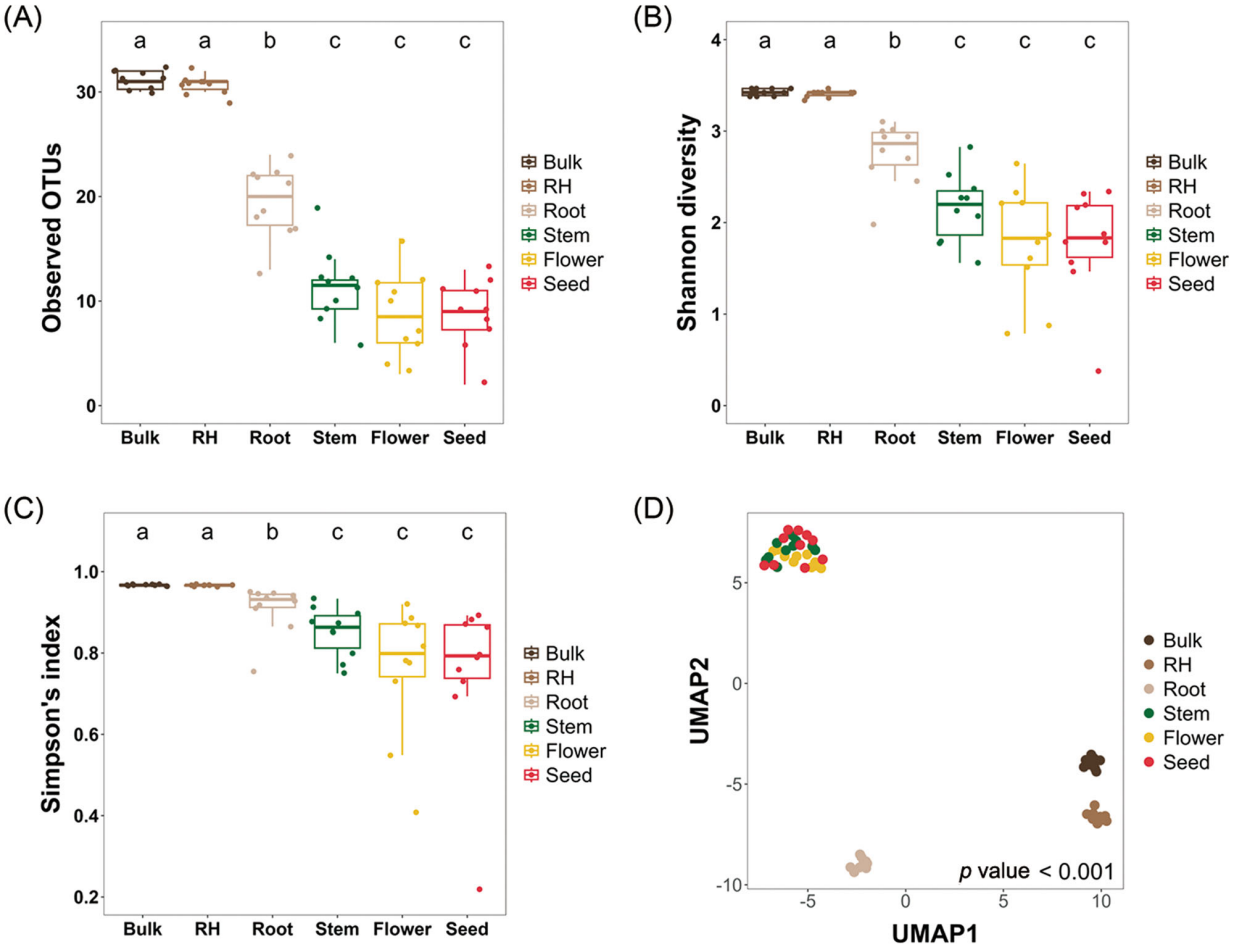
Alpha and beta diversity of bacterial communities across tomato plant compartments. (A–C) Alpha diversity across six compartments (bulk soil, rhizosphere (RH), root, stem, flower, and seed) assessed by observed OTUs (A), Shannon diversity (B), and Simpson's Index (C). Different letters above boxplots indicate significant differences among groups (Kruskal–Wallis test, *p* < 0.05). (D) Beta diversity evaluated using Bray–Curtis dissimilarity and visualized through Uniform Manifold Approximation and Projection (UMAP). Each dot represents a sample, and clustering patterns illustrate community differences across compartments. Significance was assessed by PERMANOVA (*p* < 0.001). Different letters above boxplots indicate significant differences among groups (Kruskal–Wallis test, *p* < 0.05). OUTs, operational taxonomic units; PERMANOVA, permutational multivariate analysis of variance.

Alpha diversity of bacterial communities across tomato plant compartments was assessed using the observed OTUs, Shannon index, and Simpson's index (Figure 1A–C). Bulk soil showed the highest species richness and evenness, as indicated by all three metrics, followed by the RH, which also displayed a diverse and balanced microbial community. Root samples showed reduced richness and moderate diversity, with a relatively even microbial composition compared to the above‐ground compartments. The stem and flower, and seed compartments exhibited similar levels of microbial richness (observed features), which were significantly lower than those in the bulk soil, RH, and root compartments, as shown in Figure 1A (*p* < 0.05, Kruskal‐Wallis test). The bulk soil and RH shared the highest richness (a), root showed intermediate richness (b), while stem, flower, and seed formed a group with the lowest richness (c), with no significant differences among them. Despite reduced species richness, Simpson's index suggested a balanced distribution of dominant taxa in above‐ground compartments, particularly seeds.

Beta diversity analysis (Figure 1D) revealed significant differences (*p* < 0.001) in microbial community composition across compartments. Bulk soil, RH, and roots were more dispersed, while stem, seed, and flower communities clustered together, indicating compositional similarities in above‐ground compartments.

The relative abundance analysis presented in Figure S1 revealed distinct patterns of microbial communities across different compartments of the tomato plant (bulk soil, RH, root, stem, flower, and seed). At the phylum level, bulk soil and the RH showed a rich diversity of microbial communities, with a particularly high prevalence of *Proteobacteria* (25.2% and 27.1%), while other major microbial groups including *Chloroflexi* (11.88% and 9.20%), *Planctomycetota* (10.75% and 10.55%), *Acidobacteriota* (10.41% and 9.55%), *Actinobacteriota* (10.15% and 13.83%), *Bacteroidota* (9.59% and 6.60%), and *Verrucomicrobiota* (5.72% and 5.68%) were more evenly distributed. In the root and stem compartments, the most dominant microbial phyla were *Proteobacteria* (49.44% in root; 28.11% in stem), *Firmicutes* (12.31% in root; 38.40% in stem), and *Actinobacteria* (25.38% in root; 11.65% in stem), respectively. In the flower compartment, *Firmicutes* (26.61%) and *Proteobacteria* (22.22%) dominated, displaying a distinct microbial community structure compared to other compartments. The seed compartment showed a significantly high proportion of *Firmicutes* (48.61%), indicating a unique microbial community structure compared to other parts of the plant. At the family level, bulk soil showed diverse microbial families, with notable abundances of *Pirellulaceae* (3.98%), *Chitinophagaceae* (3.88%), and *Sphingomonadaceae* (2.80%). The RH showed high relative abundances of *Gemmatimonadaceae* (4.12%), *Pirellulaceae* (3.59%), and *Chitinophagaceae* (3.46%). In the root compartment, families like *Streptomycetaceae* (15.72%), *Bacillaceae* (7.99%), and *Xanthobacteraceae* (6.81%) are prevalent, highlighting the selective environment of the root microbiota. The stem compartment included significant populations of *Bacillaceae* (31.01%), *Paenibacillaceae* (7.02%), and *Enterobacteriaceae* (6.69%), indicating a distinct microbial profile. The flower compartment was dominated by families such as *Bacillaceae* (21.06%), *Enterobacteriaceae* (5.20%), and *Neisseriaceae* (4.70%), reflecting its unique microbial environment. The seed compartment was characterized by a high abundance of *Bacillaceae* (25.42%), *Paenibacillaceae* (18.92%), and *Enterobacteriaceae* (6.09%) (Figure S1).

### Compartment‐specific taxonomic signatures identified by DESeq. 2 and Random Forest

**Figure 2:**
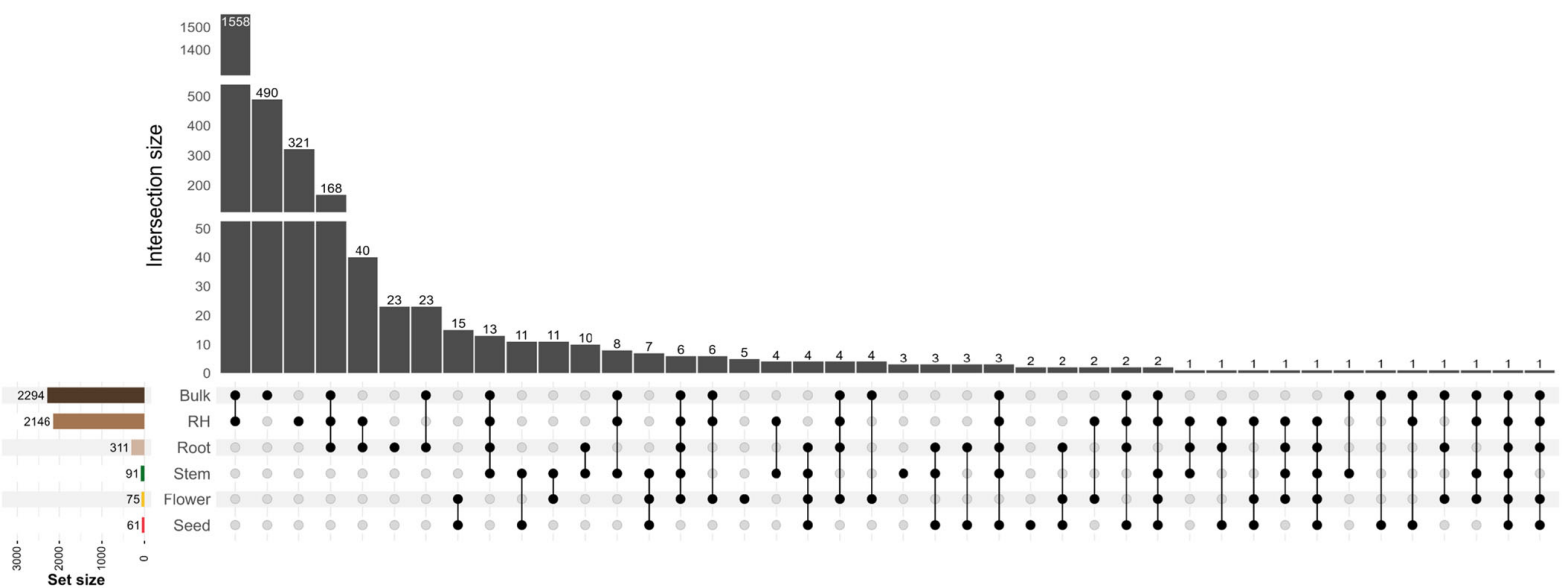
UpSet plot of overlapping OTUs across tomato plant compartments. The horizontal bars on the left represent the total number of OTUs detected in each compartment, including bulk soil, RH, root, stem, flower, and seed. The vertical bars indicate the intersection size, showing the number of OTUs shared among specific compartments. Connected dots below the bars represent the compartments involved in each intersection. This plot illustrates the unique and shared microbial communities across different tomato plant compartments, highlighting both compartment‐specific and overlapping OTUs.

**Figure 3:**
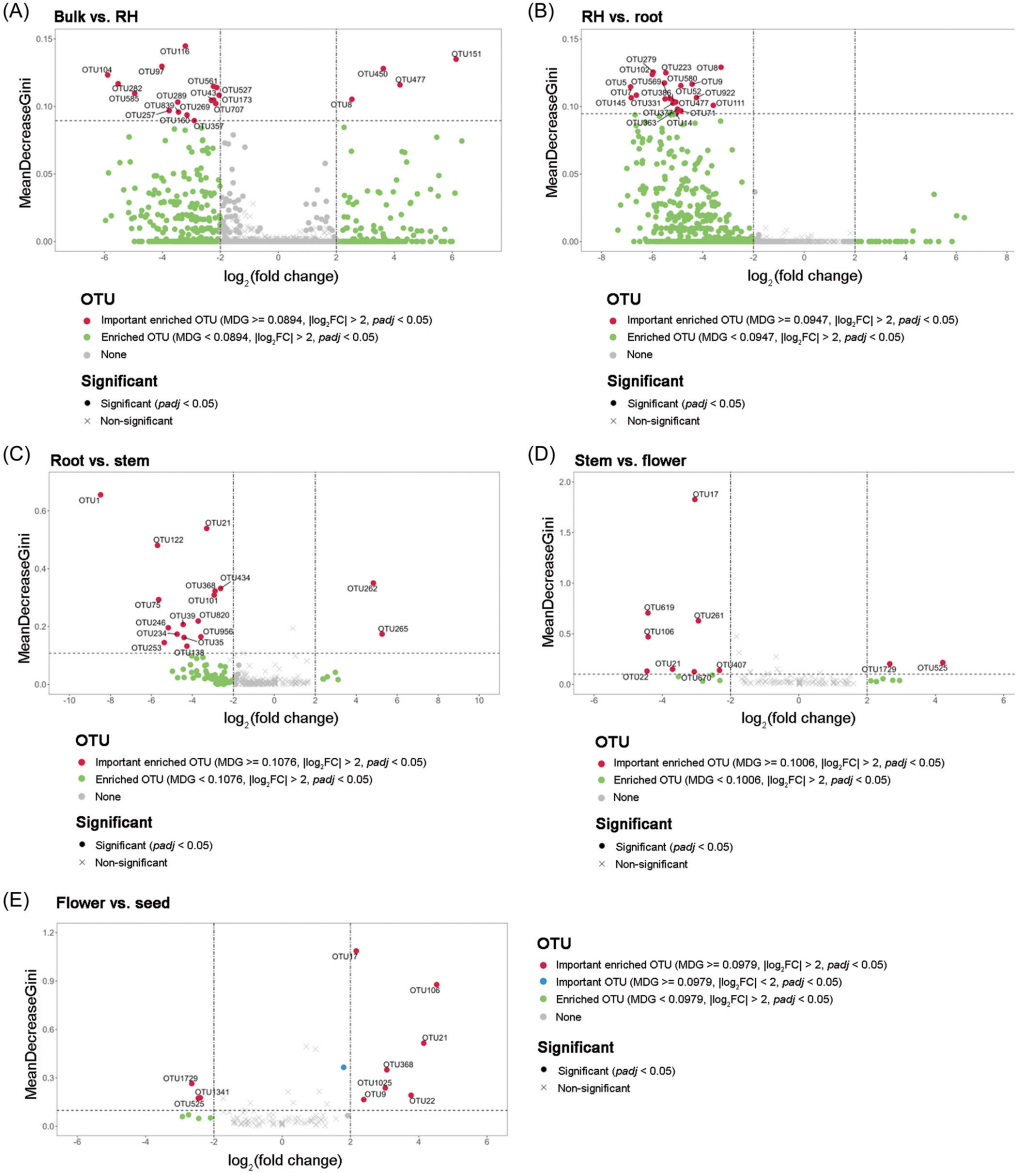
Integrated analysis of DESeq. 2 and Random Forest results to identify key OTUs. The *X*‐axis shows log_2_FC values from DESeq. 2 (absolute log_2_FC > 2) and the *Y*‐axis displays MeanDecreaseGini (MDG) scores from Random Forest (top 20 MDG). Each point represents an OTU, with the shape indicating DESeq. 2 padj significance (circles, significant; X‐marks, nonsignificant). Circle color indicates classification: red (satisfying both criteria); green (only DESeq. 2: absolute log_2_FC > 2 and *padj* < 0.05); blue (only Random Forest: top 20 MDG and *padj* < 0.05); and gray (neither condition met). FC, fold change.

We investigated the overlapping OTUs uniquely present in distinct compartments of the tomato plant using an UpSet plot (Figure 2), which revealed both unique and shared microbial taxa across compartments. Bulk soil and RH harbored the highest number of OTUs (2294 and 2146, respectively), whereas internal compartments such as stem, flower, and seed had markedly fewer OTUs (91, 75, and 61, respectively). The largest intersection involved 1558 OTUs shared across all compartments, while several smaller subsets (e.g., 490 and 321 OTUs) were shared between specific compartment combinations, indicating compartment‐specific microbial filtering and selective overlap patterns along the soil–plant continuum. Among these compartments, DESeq. 2 and Random Forest analyses were used to identify differentially abundant OTUs across plant compartments (Figure 3 and Table S1). In the bulk soil–RH comparison, 16 OTUs were enriched in bulk soil and 4 OTUs in the RH. The RH–root comparison revealed 20 OTUs enriched in the RH, while root–stem analysis showed 15 OTUs enriched in roots and 2 OTUs in stems. Similarly, the stem–flower comparison identified 8 OTUs enriched in stems and 2 OTUs in flowers. In the flower–seed transition, 3 OTUs were enriched in flowers and 7 OTUs were enriched in seeds.

Random Forest analysis supported these patterns, with OTUs showing high importance scores (MeanDecreaseGini) in distinguishing between compartments: bulk soil–RH (0.09–0.14), RH–root (0.09–0.13), root–stem (0.11–0.66), stem–flower (0.10–1.82), and flower–seed (0.10–1.09) (Figure 3 and Table S1). The complementary results from both analyses identified compartment‐specific microbial signatures, suggesting distinct selective pressures across the plant–soil interface.

### Declining microbial network complexity from soil to reproductive tissues

**Figure 4:**
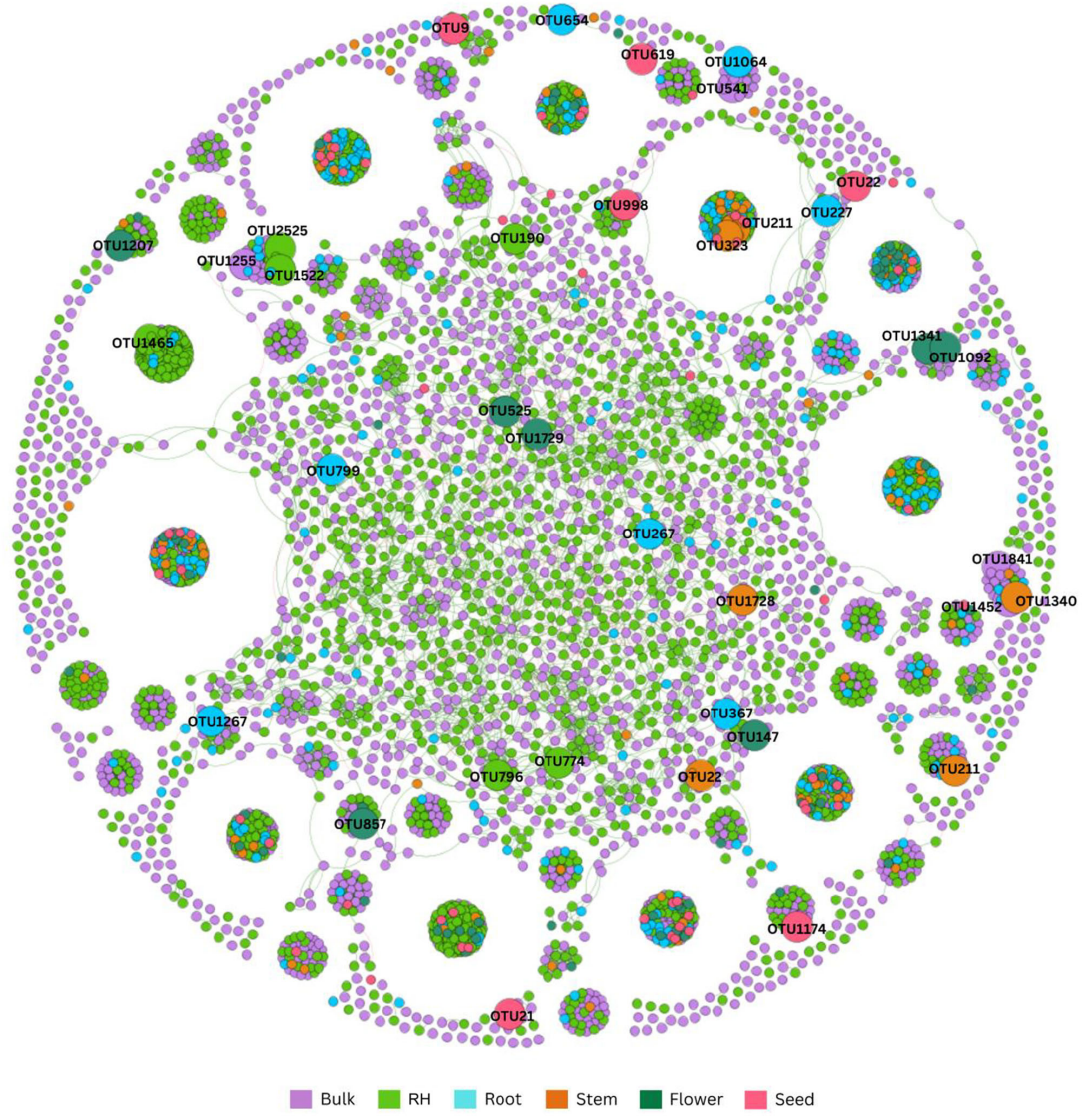
Meta‐network analysis of microbial communities across tomato plant compartments. The network shows interaction among OTUs based on degree and betweenness centrality. Each node corresponds to an OTU, and edges between nodes correspond to either positive (green) or negative (red) correlations inferred from OTU abundance profiles using the Spearman correlation (threshold = 0.95).

Meta‐network analysis (Figure 4) revealed distinct patterns of microbial interactions across plant compartments (Figure S2A–F and Table S2). The complexity of microbial networks decreased from soil to plant tissues, with the highest number of nodes and edges in bulk soil (1740 nodes, 16,330 edges) and RH (1840 nodes, 35,577 edges), followed by a sharp decline in root (308 nodes, 15,101 edges), stem (90 nodes, 1552 edges), flower (69 nodes, 873 edges), and seed (59 nodes, 430 edges) compartments (Table S2).

Network connectivity metrics showed distinct patterns: average degree was highest in root (49.029), followed by RH (19.335) and stem (17.244), while network density increased from bulk soil (0.005) to stem (0.194). The modular structure was most complex in soil compartments (bulk soil: 168 modules, RH: 106 modules) compared to plant tissues (3‐6 modules) (Table S2).

Key hub taxa were identified based on betweenness centrality and degree (Figure S2A–F). In bulk soil, five OTUs including *Latescibacterota* and *Gemmatimonas* showed high centrality (top 0.8%) and connectivity (top 13%). RH networks had six key hubs including *Actinomadura* and *Agromyces* (top 0.5% centrality, top 30% degree). Root compartment identified seven hubs including *Bacillus* and *Aeromicrobium* (top 3% centrality, top 15% degree), while stem, flower, and seed compartments showed progressively fewer but still distinct hub taxa, each characterized by specific centrality and degree thresholds (stem: top 10% and 25%; flower: top 15% and 35%; seed: top 15% and 45%, respectively).

### Soil and stem serve as major microbial sources for below‐ and above‐ground tissues

**Figure 5:**
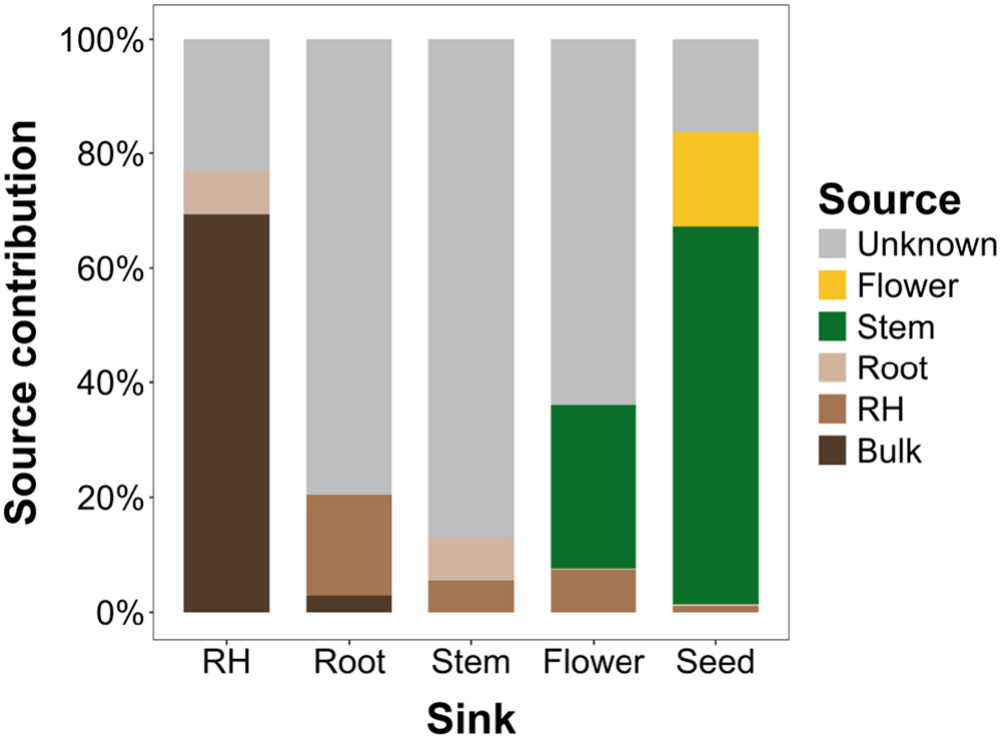
Microbial source tracking across tomato plant compartments using the fast expectation‐maximization for microbial source tracking (FEAST) algorithm. Each bar represents the average microbial source contribution to a specific compartment (RH, root, stem, flower, and seed). Colors indicate the predicted sources of microbial communities, including bulk soil, RH, and other compartments, as well as unknown sources not accounted for in the source pool.

Distinct patterns of microbial inheritance across the tomato plant were revealed through source tracking analysis (Figure 5). Below‐ground compartments were dominated by soil influence, where 69.8% of RH microbes were contributed by bulk soil. As distance from soil increased, soil‐derived microbial influence was markedly decreased and was replaced by unknown sources that were found to dominate root (81.3%) and stem (82.4%) communities. In reproductive structures, a shift toward plant‐derived sources was observed, where stem tissue was identified as a major contributor to both flower (27.8%) and seed (64.9%) microbiomes.

### Distinct ecological processes govern microbial assembly across compartments

**Figure 6:**
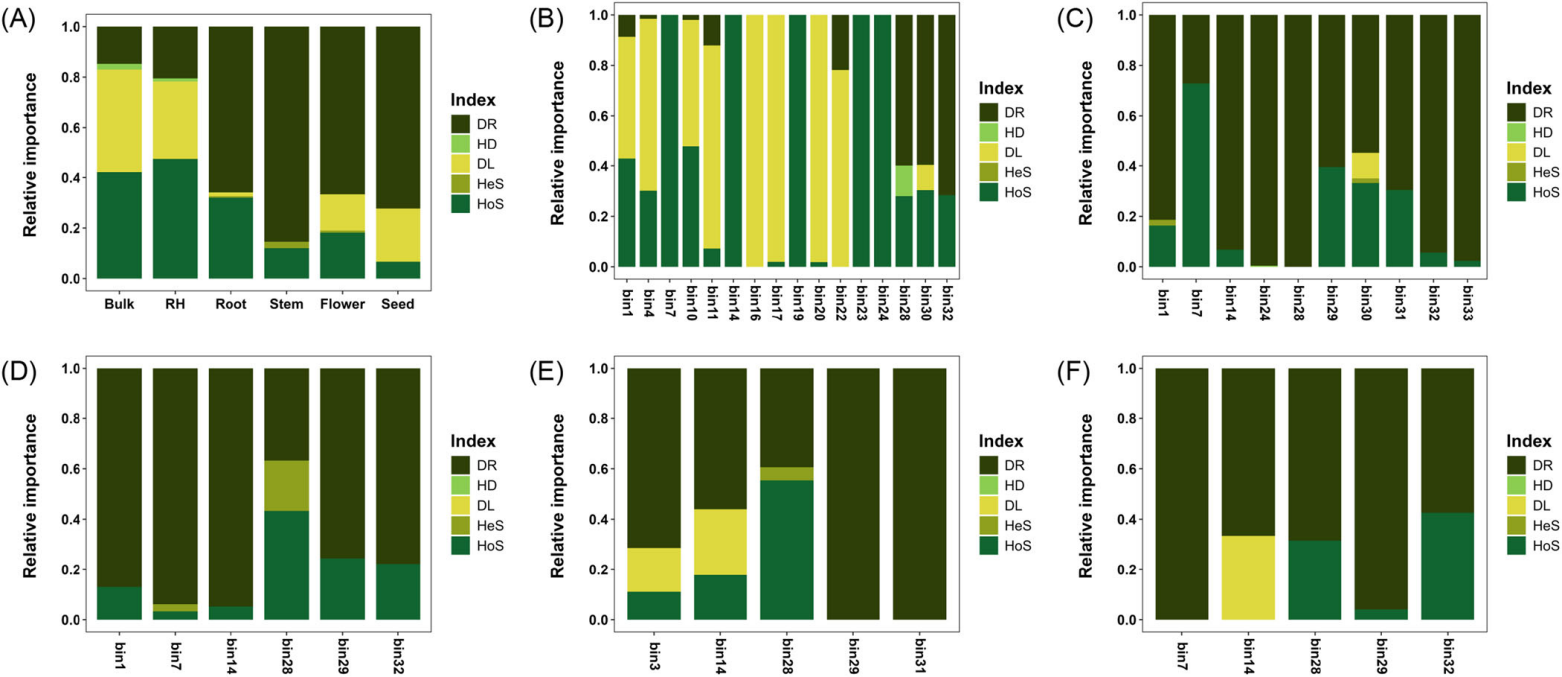
Influence of ecological processes on microbiota assembly in tomato plant compartments. (A) All compartments. (B) RH. (C) Root. (D) Stem. (E) Flower. (F) Seed. Analysis was conducted using the iCAMP R package, using beta mean pairwise distance (bMPD) as the phylogenetic metric and “Confidence” as the significance index in the icamp.big() function, to determine the dominant ecological drivers across different plant compartments. DL, dispersal limitation; DR, drift; HD, homogenizing dispersal; HeS, heterogeneous selection; HoS, homogeneous selection.

Quantitative analysis revealed distinct ecological processes dominating different plant compartments (Figure 6A–F). In the assembly processes of microbial communities in bulk soil and RH, homogeneous selection (HoS) was predominant, accounting for 42.1% and 47.3%, respectively, followed by DL at 40.8% and 30.8%, respectively. In contrast, the microbial community assembly in plant compartments was primarily governed by DR, with contributions of 65.8% in the root, 85.4% in the stem, 66.6% in the flower, and 72.2% in the seed. Additionally, HoS was the second most influential process in the root (32.0%), stem (12.0%), and flower (18.1%). However, in the seed, DL played a more prominent role compared to other plant compartments, contributing to 21.0% (Figure 6A). To investigate the ecological processes influencing enriched or network hub OTUs (key taxa) in each compartment, we analyzed the relative importance of ecological processes by phylogenetic group (bin). In the RH, key taxa were strongly influenced by HoS (5 bins out of 16 bins) and DL (9 bins out of 16 bins) (Figure 6B). The relative abundance of bins showed a positive correlation with HoS (*R* ^2^ = 0.283, *p* < 0.001) and a negative correlation with DL (*R* ^2^ = 0.244, *p* < 0.05) (Figure S3A,F). In plant compartments, key taxa were primarily associated with DR across all compartments (root: 10 bins, stem: 6 bins, flower: 5 bins, seed: 5 bins) (Figure 6C–F). Additionally, the relative abundance of bins showed a nonlinear pattern with DR (root: *R* ^2^ = 0.149, *p* < 0.05; stem: *R* ^2^ = 0.767, *p* < 0.001; flower: *R* ^2^ = 0.901, *p* = 0.001) (Figure S3G–I). However, it was not significant in the seed (*R* ^2^ = 0.994, *p* = 0.0561) (Figure S3J). HoS, the second most influential process in plant microbial communities, also showed a nonlinear relationship with the relative abundance of phylogenetic bins (root: *R* ^2^ = 0.757, *p* < 2e−16; stem: *R* ^2^ = 0.59, *p* < 0.01; flower: *R* ^2^ = 0.864, *p* < 0.001) (Figure S3B–D). Similarly, DL, the second most influential process in seed microbial community assembly, showed a strong correlation with microbial abundance (*R* ^2^ = 1, *p* < 2e−16) (Figure S3E).

## DISCUSSION

### Compartment‐specific patterns of microbial diversity and filtration

This study reveals distinct patterns of microbial diversity along the soil‐to‐plant continuum, shaped by selective filtration and dispersal processes. Bulk soil and RH compartments showed the highest microbial richness, while internal plant compartments (root, stem, flower, and seed) harbored progressively constrained communities (Figure 1A–C). These patterns reflect a selective filtration effect, where plants permit only specific taxa to infiltrate and colonize internal niches^15^, ^16^. Beta diversity analysis further highlighted unique community compositions across compartments, driven by contrasting ecological pressures between soil and aerial plant environments (Figure 1D). This gradient in microbial diversity emphasizes the interplay between deterministic selection and environmental constraints.

### Vertical transmission and uncharacterized sources shape microbial inheritance

Distinct patterns of microbial inheritance across the tomato plant highlight the selective and compartment‐specific assembly of microbial communities in response to both biotic and abiotic factors. The dominance of unknown sources (Figure 5) in internal plant compartments warrants further discussion. These unknown sources likely represent taxa that are underrepresented in current microbial reference databases or originate from niche‐specific recruitment mechanisms driven by plant hosts. For instance, plants may selectively recruit microbes based on specific metabolic or ecological traits that enhance stress tolerance, nutrient acquisition, or pathogen resistance^17^, ^18^. Additionally, stochastic processes and interactions with rare or transient microbes in the soil or phyllosphere may contribute to these uncharacterized taxa.

In reproductive structures, the shift toward plant‐derived sources underscores the role of vertical microbial transmission. Stem tissue was identified as a major contributor to flower (27.8%) and seed (64.9%) microbiomes, suggesting that microbes colonizing early vegetative tissues may be pivotal for establishing the microbial communities in later developmental stages. This finding aligns with studies highlighting the significance of plant‐mediated inheritance in shaping microbial communities across generations^19^, ^20^.

The interplay between deterministic and stochastic processes likely explains the observed transition from soil‐dominated to unknown or plant‐derived sources. Deterministic selection by the host plant may favor taxa with traits beneficial for internal colonization, whereas stochastic DR could influence community composition in less competitive niches such as stems and seeds^18^, ^21^. Future studies integrating functional genomic and metabolic profiling of these unknown taxa will be critical to elucidating their roles in plant health and microbiome dynamics.

### Compartmentalized ecological processes govern microbial community assembly

Microbial community assembly across compartments was shaped by a combination of deterministic and stochastic processes, with distinct ecological drivers dominating in different niches. DL was the primary process in soil‐associated compartments, such as bulk soil and RH, where environmental heterogeneity and physical barriers limited microbial movement^22^, ^23^. Additionally, DL showed a negative correlation with the relative abundance of phylogenetic groups, which indicates that rare taxa are affected by DL and highly constrained in its movement to other surrounding environments, such as bulk soil and root (Figure S3F). Particularly, *Thermomicrobiales* (Bin11), *Verrucomicrobiae* (Bin16 and Bin17), *Planctomycetota* (Bin20 and Bin22) were enriched in RH than root (Figure 6B, Supplementary Information and Table S1). Several groups of microbes abundantly present in the RH provide beneficial effects to plants and may be dependent on root exudates, limiting their dispersal from the RH to other environments.

In contrast, stochastic DR dominated plant‐associated compartments, particularly roots and stems, where community assembly was less influenced by environmental selection (Figure 6C–F). This shift from deterministic to stochastic processes along the soil‐to‐plant continuum aligns with the conceptual synthesis of community assembly processes provided by Vellend^21^, stochastic niche theory^8^, and neutral theory^7^. Additionally, correlations between DR and the relative abundance of phylogenetic groups tended to saturate the proportion of DR with an increase in its relative abundance (Figure S3G,I,J). This suggests that stochastic DR in microbial communities in plant compartments is not simply a random process, but another process affected by various factors.

While less dominant, deterministic processes, including homogenizing dispersal (HD) and HoS, played roles in specific compartments. HD was observed only in bin 28 in the RH (Figure 6B), where microbial connectivity may have facilitated limited homogenization. This observation aligns with Stegen et al.^24^, who demonstrated that high dispersal rates reduce community differentiation in connected environments. Similarly, HoS was prominent in select bins, such as bins 23 and 24 in the RH, bin 7 in roots, bin 28 in stems and flowers, and bins 28 and 32 in seeds (Figure 6B–G). These findings are in agreement with those reported by Hanson et al.^25^, who emphasized that stable environmental conditions and uniform selection pressures favor specific taxa, leading to deterministic patterns of community assembly. Together, these findings highlight the importance of balancing multiple ecological drivers in structuring microbial communities across heterogeneous environments.

### Ecological hub taxa play functional roles in plant health

Network analysis identified several taxa as critical ecological hubs across compartments, contributing to plant health and resilience. *Aeromicrobium* (23.5% of the root microbial community) and *Bacillus* spp., present in both roots and seeds, play roles in nutrient cycling, disease suppression, and plant growth promotion^26^, ^27^. In the stem and seed compartments, taxa such as *Corynebacterium* sp., *Sediminibacterium* sp., and *Aquabacterium* sp. were identified as key players in stress mitigation and nutrient mobilization^28^, ^29^, ^30^.

Bee‐mediated microbes, including *Gilliamella apis*, observed in the flower compartment, highlight the influence of external biotic factors on plant microbiota^31^, ^32^. These interactions suggest that microbial transfer via pollinators can shape microbial communities in aerial plant compartments. Together, these findings underscore the functional and ecological significance of microbial hubs within the tomato plant holobiont.

### Biotic and abiotic interactions define the tomato plant holobiont

The interplay of ecological drivers, microbial taxa, and biotic interactions highlights the complexity of plant–microbe interactions. Leveraging beneficial microbes, such as *Bacillus* spp. for nutrient solubilization and *Aquabacterium* for stress mitigation, presents opportunities for sustainable agriculture^10^. Targeted microbiome management strategies, informed by ecological theory, can enhance crop health, resilience, and productivity.

While this study provides valuable insights, the absence of environmental and functional data limits the mechanistic understanding of microbial community assembly. Factors such as soil chemistry, climatic conditions, and plant traits likely influenced microbial dynamics but were not measured. Future studies should integrate these variables, alongside functional genomic and metabolic analyses, to unravel the roles of microbial taxa and ecological drivers. Such approaches will complement the ecological insights provided here and enable the targeted manipulation of plant microbiomes for improved crop production.

In summary, this study reveals a gradient of microbial diversity from soil to plant compartments in tomato plants, driven by distinct ecological drivers. Deterministic processes, such as DL, dominate soil compartments, while stochastic DR shapes plant tissues. The hub taxa such as *Aeromicrobium, Bacillus*, and *Aquabacterium* identified through co‐occurrence network analysis, contribute to plant health, while bee‐mediated microbes highlight external biotic influences on aerial microbiomes. These findings offer a framework for leveraging beneficial microbes to enhance crop productivity and resilience, with future research focusing on environmental and functional data to advance microbiome management for sustainable agriculture.

## MATERIALS AND METHODS

### Tomato plant cultivation and sampling

Tomato seeds (*Solanum lycopersicum* cv. Hawaii 7996) were germinated on filter paper with sterilized distilled water for 1 week, and then grown in general‐purpose soil within a growth chamber (27°C, 65% humidity, 10‐h light/14‐h dark cycle) for 4 weeks. After acclimation in a greenhouse (25–30°C) for a week, plants were transplanted into pots with a 1:1 mix of Dong‐A University field soil and general‐purpose soil to balance the nutrient content and physical structure, supporting optimal plant growth and mimicking typical agricultural conditions.

At ten weeks, samples (*n* = 10 per compartment) were collected from bulk soil, RH, root, stem, and seed, while flowers (*n* = 10) were collected at 6 weeks. Bulk soil was obtained after surface debris was removed, and RH soil was collected by shaking roots and centrifugation. Roots, stems, and seeds were excised using sterile tools, and flowers were collected using sterile gloves.

### Sample preparation and DNA extraction

Tomatoes were surface‐sterilized (90% ethanol), and seeds were extracted, sterilized (70% ethanol, 2.5% bleach), and rinsed. Stems and roots were cleaned [1× TE buffer (1 mM, pH 8.0 EDTA + 10 mM, pH 8.0 Tris‐Cl)], rinsed [Triton X‐100 (0.1%, v/v)], sterilized (80% ethanol, 3% bleach), rinsed (SDW) again, and stored at −80°C. DNA was extracted from seeds, flowers, stems, and roots using the CTAB method, and from bulk and RH soil using the FastDNA® Spin Kit (MP Biomedicals) for Soil, following manufacturers’ protocols.

### 16S rRNA sequencing workflow and data processing

To amplify the V3–V4 hypervariable regions of the 16S rRNA gene, universal primers 341F (5′‐TCGTCGGCAGCGTCAGATGTGTATAAGAGACAGCCTACGGGNGGCWGCAG‐3′) and 805R (5′‐GTCTCGTGGGCTCGGAGATGTGTATAAGAGACAGGACTACHVGGGTATCTAATCC‐3′), each containing Illumina overhang adaptor sequences, were used. To prevent plant mitochondrial and plastid DNA contamination, peptide nucleic acid PCR blockers (PNA clamps) were added during the first PCR. The amplification profile used the following conditions: initial denaturation at 95°C for 3 min, followed by 25 cycles of denaturation at 95°C for 30 s, annealing at 55°C for 30 s, and extension at 72°C for 30 s, with a final extension step at 72°C for 5 min. The amplicons were subjected to clean‐up by the Agencourt AMPure XP PCR Purification system (Beckman Coulter) to remove PCR primers and primer dimers. Second, library preparation and sequencing were performed at NICEM (National Instrumentation Center for Environmental Management), Seoul National University in Seoul, Korea. The libraries were sequenced on the Illumina MiSeq platform (Illumina, Inc.). The DADA2 (Divisive Amplicon Denoising Algorithm) plug in the QIIME2 (version 2022.02) pipeline was used for low‐quality sequence removal, chimeric sequence removal, and merging of forward and reverse sequences. Using the de novo VSEARCH (v2.7.0) algorithm (vsearch cluster‐features‐de‐novo)^33^, the 97% pairwise nucleotide sequence threshold was used for clustering merged sequences into OTUs (Supplementary Information). The taxonomy of OTUs was assigned using the Naïve Bayes algorithm implemented in the q2‐feature‐classifier prefitted to the SILVA database (version 138.1) for the V3–V4 region of 16S rRNA gene (Supplementary Information).

### Alpha diversity, beta diversity, and relative abundance analysis

For the analysis of alpha diversity, the rarefy_even_depth function in phyloseq (v1.48.0) R package (v4.3.1)^34^ was used to rarefy the sequencing depths by subsampling the OTU table. We quantified bacterial alpha diversity by calculating observed OTUs, the Shannon index, and Simpson's index using the rarefied OTU table. Beta diversity was evaluated using Bray–Curtis dissimilarity measures on a normalized OTU table. The statistical significance of beta diversity was determined using PERMANOVA (permutational multivariate analysis of variance), with 999 permutations to ascertain significance levels. The analysis of relative abundance was performed using the MicrobiotaProcess (v1.14.1) package in R, which normalized the OTU counts to reflect the relative abundance of each OTU in the samples.

### DESeq. 2, Random Forest, and network analyses

In this study, we applied the DESeq. 2 package to detect significant differences in taxa across the compartments. DESeq. 2 was used with a negative binomial model and significance was determined using the Wald test. Adjustments for false discovery rates were made using the Benjamini–Hochberg method, considering FDR‐adjusted *p*‐values below 0.05 as significant. For univariate analysis, the randomForest package provided supervised classification models to estimate microbial richness and relative abundance. This machine learning approach identified key predictors, assessing their importance via the MeanDecrease Gini (MDG) across a forest of 100 trees using OTUs as predictors, in line with the methodologies of Liaw^35^ and Breiman^36^. Network analysis was conducted to explore differences in the microbiome of different compartments, examining relative OTU abundances. This analysis used data from 10 biological replicates, constructing networks from OTUs present in at least 70% of samples. The Random Matrix Theory (RMT) was used to set the similarity threshold for network connections, based on the criteria described by Deng et al.^37^. All network analyses were performed with the Molecular Ecological Network Analyses (MENA) Pipeline (http://ieg2.ou.edu/MENA/ou)^37^. Network visualization was executed using Cytoscape 3.10.2^38^ and Gephi 0.10.1.

### Analyses of assembly mechanisms and microbial source tracking

To discern the ecological processes shaping the microbiota assembly at the OTU level within each compartment, we utilized the iCAMP R package^39^. The beta mean pairwise distance (bMPD) served as the phylogenetic metric, while ‘Confidence’ was used as the significance index parameter within the icamp.big() function. The icamp.big() function specifically calculates the contributions of various ecological processes, including HoS, heterogeneous selection (HeS), DR, DL, and HD, to community assembly. This approach enabled the identification of the prevailing ecological drivers influencing microbial distribution within each plant compartment. Furthermore, correlations between the relative importance of ecological processes and the relative abundance of phylogenetic group (bin) in each compartment were analyzed by generalized additive modeling (GAM) using the mgcv (v1.8‐42) R package.

To understand the roles of plant compartments as both microbial sources and sinks, we used the fast expectation maximization for the microbial source tracking (FEAST) algorithm^40^. This method quantified microbial contributions from bulk soil, RH, and other compartments, providing insights into microbial dispersal and assembly mechanisms.

### Statistical analysis

Shapiro–Wilk tests were performed to evaluate the normality of the data, and Levene's test was utilized to assess the homogeneity of variances using the lawstat package^41^. For alpha‐diversity analysis, Kruskal–Wallis one‐way analysis of variance was used. In the Simpson analysis, pairwise comparisons were conducted using Wilcoxon rank sum tests to obtain *p*‐values. The comparisons included bulk soil vs. RH soil (Bulk‐RH), RH soil vs. root (RH‐root), root vs. stem (root–stem), stem vs. flower (stem–flower), and flower vs. seed (flower–seed).

## AUTHOR CONTRIBUTIONS

**Nazish Roy**: Formal analysis; validation; writing—original draft. **Seongeun Yang**: Data curation; formal analysis; investigation; visualization; writing—review and editing. **Dongmin Lee**: Formal analysis; investigation; methodology; validation; visualization; writing—review and editing. **Kihyuck Choi**: Conceptualization; funding acquisition; investigation; project administration; resources; writing—original draft; writing—review and editing.

## CONFLICT OF INTERESTS

The authors declare no conflict of interests.

## ETHICS STATEMENT

This study did not involve human participants or animal experimentation, and therefore, did not require institutional ethics approval. All procedures involving plant material were conducted in accordance with institutional and national guidelines for research integrity and ethical standards.

## Supporting information


**Figure S1.** Relative abundance of microbial communities in tomato plant compartments at different taxonomic levels: (A) Phylum and (B) Family distributed across six compartments: Bulk, RH, Root, Stem, Flower, and Seed.

**Figure S2.** Network centrality analysis of microbial operational taxonomic units (OTUs) across different compartments. Each subplot represents a scatter plot correlating the degree and betweenness centrality of OTUs within a specific plant compartment: (A) Bulk, (B) RH, (C) Root, (D) Stem, (E) Flower, and (F) Seed. The degree of each OTU is plotted on the *x*‐axis and betweenness centrality is plotted on the *y*‐axis. OTUs with a degree and betweenness centrality in the top percentage for their respective compartment are indicated in red. Thresholds for top‐performing OTUs vary by compartment, reflecting the compartment‐specific importance of certain microbes: (A) Betweenness centrality: top 0.8%, Degree: top 13%, (B) Betweenness centrality: top 0.5%, Degree: top 30%, (C) Betweenness centrality: top 3%, Degree: top 15%, (D) Betweenness centrality: top 10%, Degree: top 25%, (E) Betweenness centrality: top 15%, Degree: top 35%, (F) Betweenness centrality: top 15%, Degree: top 45%. The dashed lines delineate these top‐performing thresholds for betweenness centrality (horizontal) and degree (vertical) within each plot.

**Figure S3**. Correlation analysis between the relative abundance of phylogenetic bins and the relative importance of ecological drivers. The *X*‐axis represents the relative abundance of each bin in individual compartments and the *Y*‐axis depicts the relative importance of ecological drivers associated with each bin. Analysis was performed using the gam() function in the mgcv R package, applying generalized additive modeling (GAM) to detect potential nonlinear relationships. The grey area indicates the 95% confidence interval, while each circle represents an individual bin.

**Sheet 1: Illumina_experimental design**: Sample ID, compartment, and description of each sample collected from different compartments of the tomato plant. **Sheet 2: Illumina_OTU table**: The OTU table derived from Illumina sequencing data, listing OTU IDs, and their corresponding read counts across different samples. **Sheet 3: SILVA_Taxonomy_Illumina**: Taxonomic classification of the OTUs, mapping each OTU to its corresponding taxonomic groups (phylum to genus level) based on the SILVA database.

**Table S1.** OTUs identified by DESeq. 2 and Random Forest (RF) analyses across different compartments of the tomato plant: Bulk versus RH, RH versus Root, Root versus Stem, Stem versus Flower, and Flower versus Seed as significant by each method for the respective comparisons.

**Table S2.** Network properties derived from network analysis of bacterial communities across different compartments of the tomato plant.

## Data Availability

The 16S rRNA based sequencing data have been deposited under BioProject (https://www.ncbi.nlm.nih.gov/bioproject/) with accession number PRJNA1100984 and under BioSample (https://www.ncbi.nlm.nih.gov/biosample/) with accessions numbers SAMN40982745 ‐ SAMN40982804.
