# Supplementary figures and images for: Ecological processes influencing bacterial community assembly across plant niche compartments
Source: mLife. 2025 Jun 24;4(3):294–304. doi: 10.1002/mlf2.70019 (PMC12207902; doi:10.1002/mlf2.70019)

# Supplementary Fig. S1

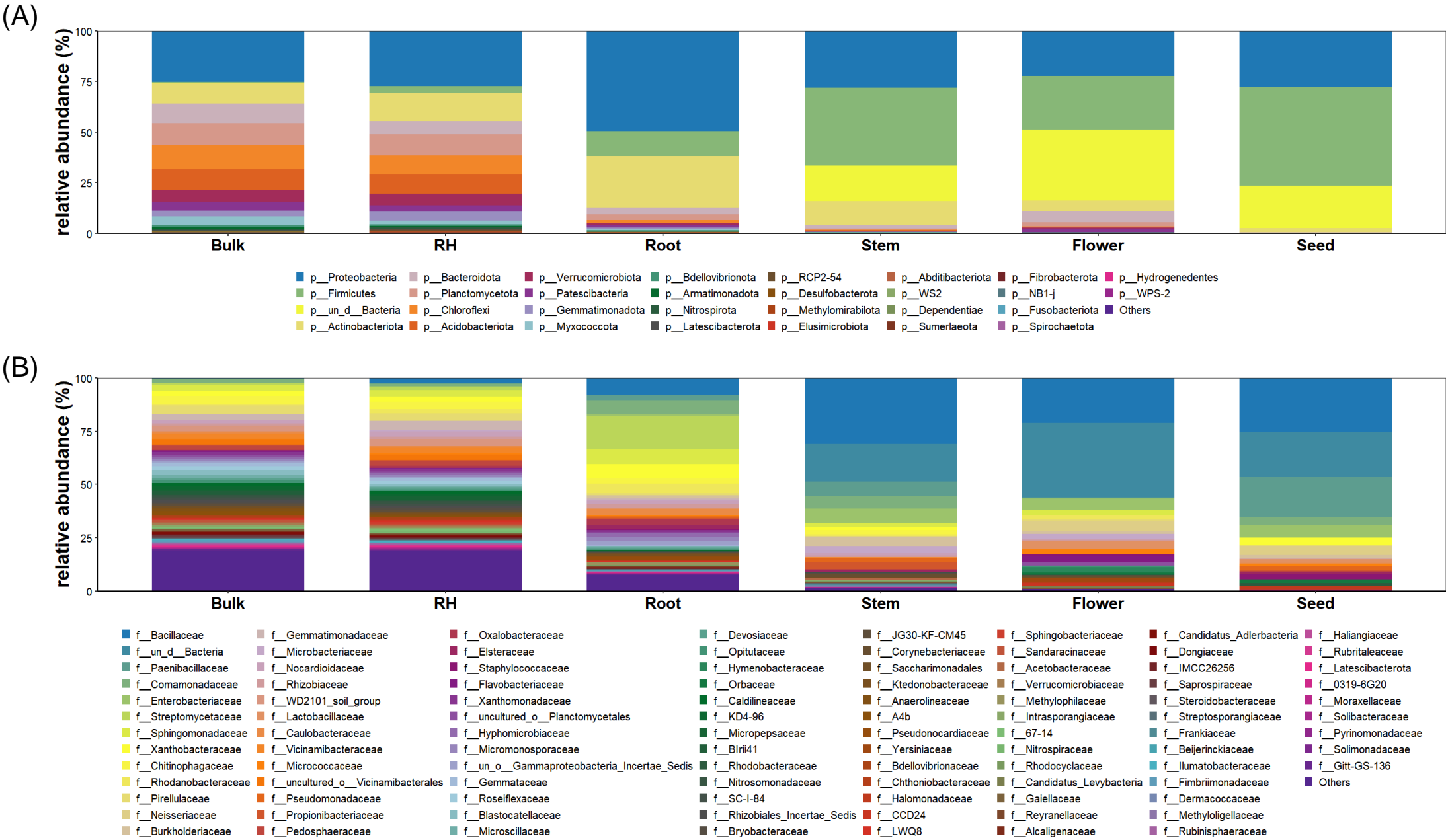

Supplement: Supplementary file 1 — Figure S1. Relative abundance of microbial communities in tomato plant compartments at different taxonomic levels: (A) Phylum and (B) Family distributed across six compartments: Bulk, RH, Root, Stem, Flower, and Seed. [file MLF2-4-294-s004.pdf]

# Supplementary Fig. S2

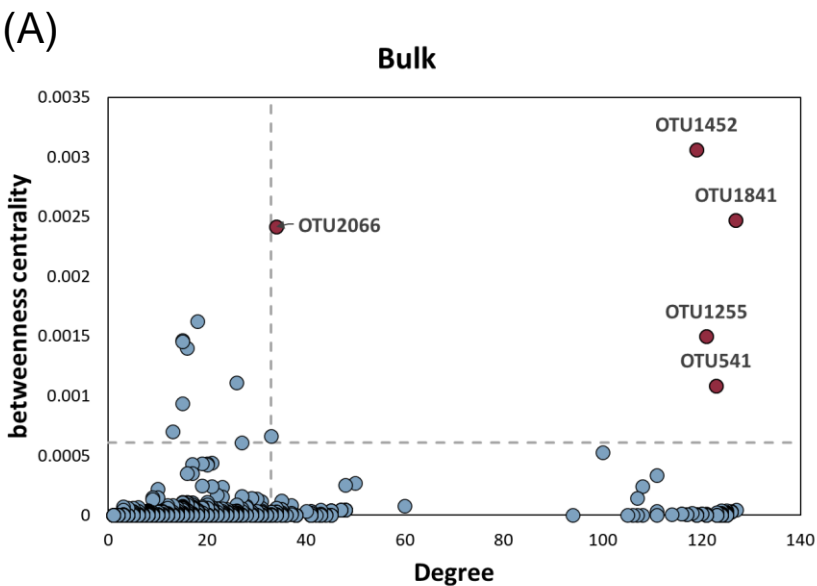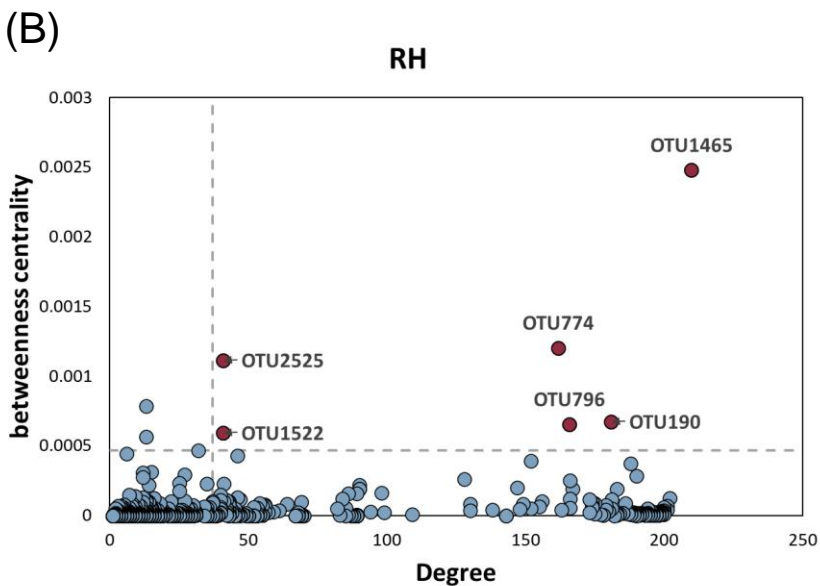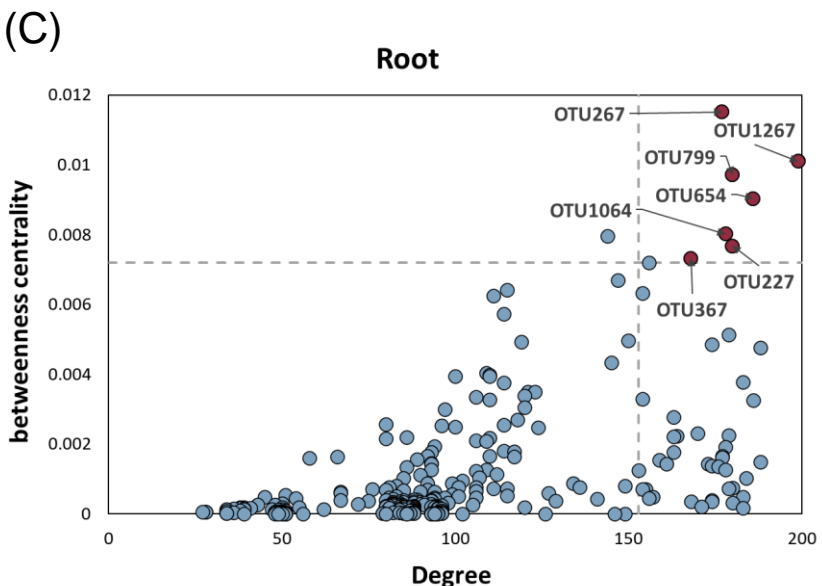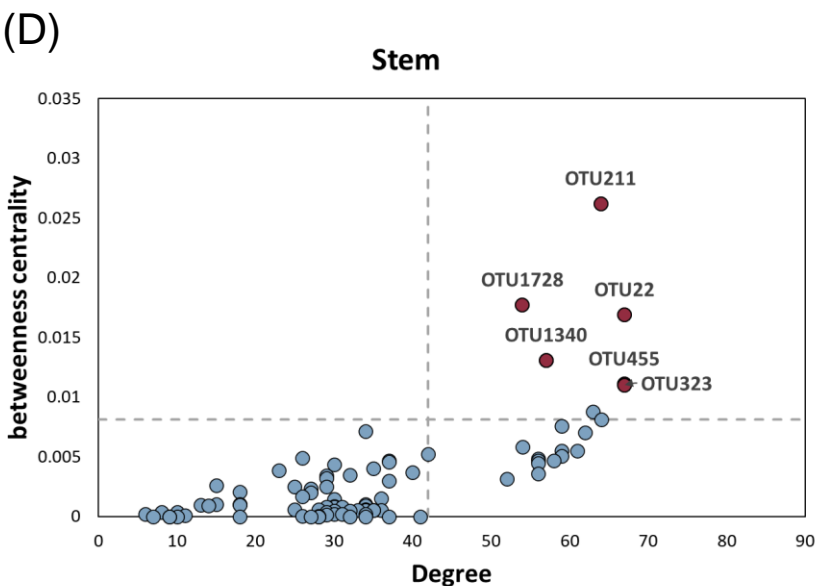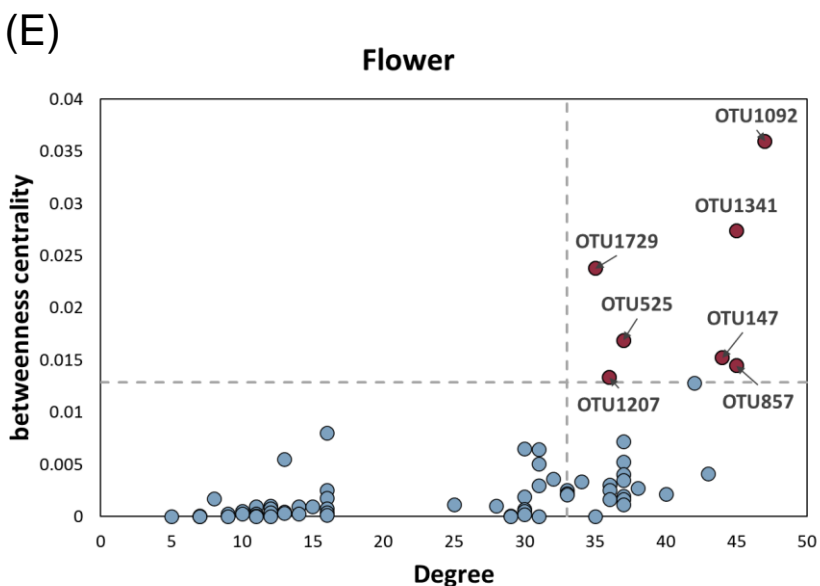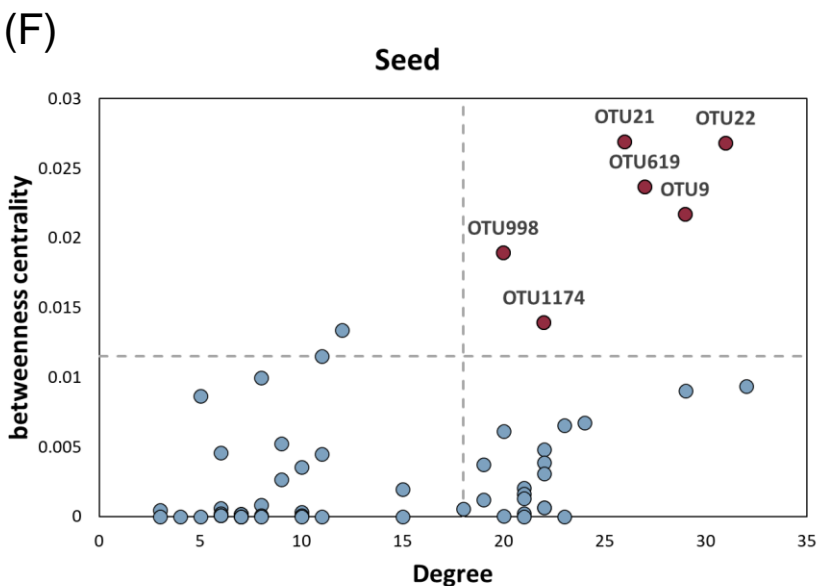

Supplement: Supplementary file 2 — Figure S2. Network centrality analysis of microbial operational taxonomic units (OTUs) across different compartments. Each subplot represents a scatter plot correlating the degree and betweenness centrality of OTUs within a specific plant compartment: (A) Bulk, (B) RH, (C) Root, (D) Stem, (E) Flower, and (F) Seed. The degree of each OTU is plotted on the x‐axis and betweenness centrality is plotted on the y‐axis. OTUs with a degree and betweenness centrality in the top percentage for their respective compartment are indicated in red. Thresholds for top‐performing OTUs vary by compartment, reflecting the compartment‐specific importance of certain microbes: (A) Betweenness centrality: top 0.8%, Degree: top 13%, (B) Betweenness centrality: top 0.5%, Degree: top 30%, (C) Betweenness centrality: top 3%, Degree: top 15%, (D) Betweenness centrality: top 10%, Degree: top 25%, (E) Betweenness centrality: top 15%, Degree: top 35%, (F) Betweenness centrality: top 15%, Degree: top 45%. The dashed lines delineate these top‐performing thresholds for betweenness centrality (horizontal) and degree (vertical) within each plot. [file MLF2-4-294-s001.pdf]

# Supplementary Fig. S3

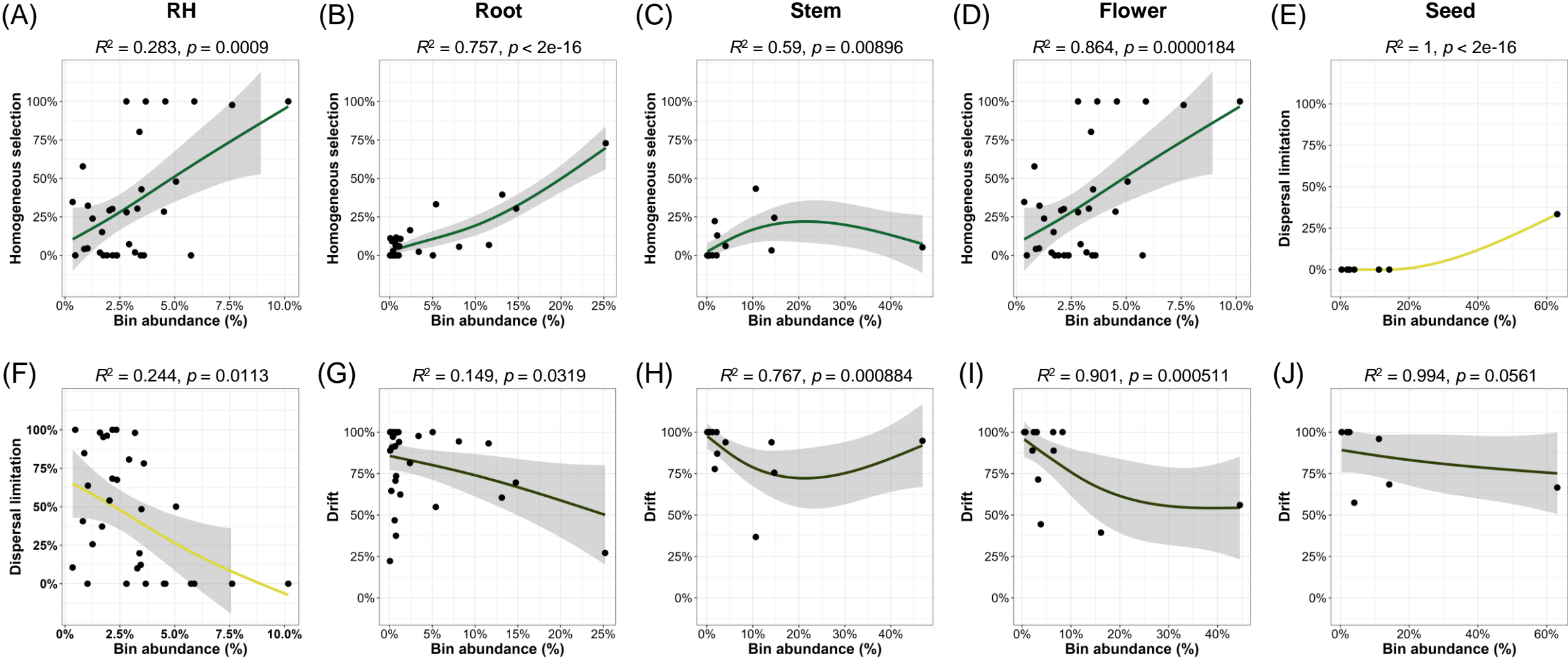

Supplement: Supplementary file 3 — Figure S3. Correlation analysis between the relative abundance of phylogenetic bins and the relative importance of ecological drivers. The X‐axis represents the relative abundance of each bin in individual compartments and the Y‐axis depicts the relative importance of ecological drivers associated with each bin. Analysis was performed using the gam() function in the mgcv R package, applying generalized additive modeling (GAM) to detect potential nonlinear relationships. The grey area indicates the 95% confidence interval, while each circle represents an individual bin. [file MLF2-4-294-s003.pdf]
