# Supplementary material for: Ecological processes influencing bacterial community assembly across plant niche compartments
Source: mLife. 2025 Jun 24;4(3):294–304. doi: 10.1002/mlf2.70019 (PMC12207902; doi:10.1002/mlf2.70019)
Supplement: Supplementary file 6 — Table S2. Network properties derived from network analysis of bacterial communities across different compartments of the tomato plant. [file MLF2-4-294-s002.docx]

Supplementary Table S2.

| Network properties | Bulk | RH | Root | Stem | Flower | Seed |
| --- | --- | --- | --- | --- | --- | --- |
| Total nodes ^a^ | 1,740 | 1,840 | 308 | 90 | 69 | 59 |
| Total edges ^b^ | 16,330 | 35,577 | 15,101 | 1,552 | 873 | 430 |
| Average Degree ^c^ | 9.385 | 19.335 | 49.029 | 17.244 | 12.652 | 7.288 |
| Network Diameter ^d^ | 14 | 10 | 5 | 5 | 4 | 6 |
| Network Density ^e^ | 0.005 | 0.011 | 0.16 | 0.194 | 0.186 | 0.126 |
| Average Clustering Coefficient ^f^ | 0.311 | 0.378 | 0.293 | 0.305 | 0.313 | 0.297 |
| Average Path Length ^g^ | 3.234 | 2.145 | 1.708 | 1.615 | 1.618 | 1.807 |
| The number of module^h^ | 168 | 106 | 6 | 3 | 3 | 4 |

^a^ Microbial taxa with at least one significant correlation

^b^ Number of connections/correlations generated by MENA analysis

^c^ The average number of connections per node in the network, that is, the node connectivity (Gephi).

^d^ The longest distance between nodes in the network, measured in number of edges (Gephi).

^e^ Measure how close the network is to complete (Gephi).

^f^ How nodes are embedded in their neighborhood and the degree to which they tend to cluster together (Gephi).

^g^ Average network distance between all pair of nodes or the average length off all edges in the network (Gephi).

^h^ The presence of different groups of nodes with high number of edges (correlations) within and with some degree of independencies between groups
